# Long-term neuronal survival, regeneration, and transient target reconnection after optic nerve crush and mesenchymal stem cell transplantation

**DOI:** 10.1186/s13287-019-1226-9

**Published:** 2019-04-17

**Authors:** Louise A. Mesentier-Louro, Leandro C. Teixeira-Pinheiro, Fernanda Gubert, Juliana F. Vasques, Almir J. Silva-Junior, Luiza Chimeli-Ormonde, Gabriel Nascimento-dos-Santos, Rosalia Mendez-Otero, Marcelo F. Santiago

**Affiliations:** 1 Instituto de Biofísica Carlos Chagas Filho, Universidade Federal do Rio de Janeiro, Rio de Janeiro, RJ 21941-902 Brazil; 2 National Institute of Science and Technology for Regenerative Medicine-REGENERE, Rio de Janeiro, Brazil

## Abstract

**Background:**

Retina and/or optic nerve injury may cause irreversible blindness, due to degeneration of retinal ganglion cells. We and others have previously shown that the intravitreal injection of mesenchymal stem cells (MSCs) protects injured retinal ganglion cells and stimulates their regeneration after optic nerve injury, but the long-term effects of this therapy are still unknown.

**Methods:**

We injected rat MSC (rMSC) intravitreally in adult (3–5 months) Lister Hooded rats of either sex after optic nerve crush. Retinal ganglion cell survival, axonal regeneration, and reconnection were analyzed 60 and 240 days after crush by immunohistochemistry for Tuj1, anterograde labeling with cholera-toxin B and by immunohistochemistry for nerve growth factor-induced gene A (NGFI-A, driven by light stimulation) in the superior colliculus after a cycle of light deprivation-stimulation. Visual behaviors (optokinetic reflex, looming response, and preference for dark) were analyzed 70 days after crush.

**Results:**

rMSC treatment doubled the number of surviving retinal ganglion cells, preferentially of a larger subtype, and of axons regenerating up to 0.5 mm. Some axons regenerated to the lateral geniculate nucleus and superior colliculus. NGFI-A+ cells were doubled in rMSC-treated animals 60 days after crush, but equivalent to vehicle-injected animals 240 days after crush, suggesting that newly formed synapses degenerated. Animals did not recover visual behaviors.

**Conclusions:**

We conclude that rMSC-induced neuroprotection is sustained at longer time points. Although rMSCs promoted long-term neuroprotection and long-distance axon regeneration, the reconnection of retinal ganglion cells with their targets was transitory, indicating that they need additional stimuli to make stable reconnections.

**Electronic supplementary material:**

The online version of this article (10.1186/s13287-019-1226-9) contains supplementary material, which is available to authorized users.

## Introduction

The visual information is conveyed from the eye to the brain through the axons of retinal ganglion cells (RGCs), which form the optic nerve. Diseases that affect the retina and the optic nerve, such as glaucoma and ischemic optic neuropathies, may lead to irreversible blindness [1]. Furthermore, the retina and the optic nerve have long been used to study the central nervous system (CNS) regeneration, harboring a simpler system than the brain and spinal cord [2].

Optic nerve regeneration can be experimentally induced by different approaches, such as by delivering neurotrophic factors [3, 4], increasing ocular inflammation [5–8] and manipulating genes targeting growth-related inhibitors such as phosphatase and tensin homolog (PTEN), Kruppel-like family (KLF) transcription factors, and the suppressor of cytokine signaling 3 (SOCS3) [9–11]. The regeneration in PTEN knockout mice is due to the activation of the mammalian target of rapamycin (mTOR), which can be stimulated by different strategies, leading to extensive axonal regeneration and partial functional recovery only when combined with other approaches [12–15]. Interestingly, many of these pro-regenerative pathways are at least indirectly associated with tumor growth, raising concern about the clinical feasibility of their manipulation [16]. In addition, complex combinatorial approaches are still far from translation.

Our group showed that intravitreally injected bone marrow mononuclear cells (BMMCs) promote RGC survival and regeneration after optic nerve crush [17]. Because RGC survival declined over time, we continued our studies using mesenchymal stem cells (MSCs) [18], which were neuroprotective in a glaucoma model [19]. Although MSCs sustained neuroprotection for at least 28 days after crush (d.a.c.) [18], the long-term fate of RGCs, potential target reconnection, and functional recovery are still unknown.

MSCs are the prevalent cell type in NIH clinical trials [20] and the transplantation of bone marrow-derived cells indicated safety and feasibility in models of neurological diseases [21–23]. However, understanding MSC effects is important to avoid unsuccessful trials, as the transplantation of cells into the eye without supporting pre-clinical data and appropriate procedures can lead to severe visual loss [24].

We found that MSCs promoted long-term neuroprotection, long-distance axon regeneration, and synaptic reconnection after optic nerve crush. However, RGC reconnection with their targets was transitory, indicating that RGCs need additional stimuli to make stable reconnections.

## Materials and methods

### Experimental design and statistics

Adult Lister Hooded rats (3–5-month-old, both sexes) were used according to protocols approved by the Committee for the Use of Experimental Animals from the Federal University of Rio de Janeiro (#IBCCF177). Effort was made to minimize suffering and to perform as many as possible *post-mortem* analysis using the same animal, reducing the number of animals.

Animals underwent unilateral optic nerve crush with (1) rMSC or (2) vehicle injection. The contralateral eyes were used as control, except for visual behavior. Four to ten animals were included per group and time point, based on our previous studies [17, 18, 21, 25–27]. Animals are randomized per group, and all quantifications were performed by masked observers. Animals with lens injury were excluded from the study.

Data was tested for normality using D’Agostino Pearson test and analyzed using parametric or non-parametric tests based on the type of distribution (normal or non-normal, respectively). Comparisons between two groups were performed using an unpaired *t* test (parametric) or Mann-Whitney (non-parametric), depending on the result of the normality test; three or more groups were compared using one-way analysis of variance with Tukey’s multiple comparisons test, which was chosen to compare every mean with every other mean considering the scatter from all groups to perform multiple comparisons with adjusted *P* value, assuming that all groups are samples from populations with same standard deviation. For grouped analyses (axon regeneration and white-black box test in naïve animals), we performed two-way ANOVA with Holm-Sidak’s method to correct for multiple comparisons. Prism 6.0 (GraphPad, San Diego, CA, USA) was used for all statistical analyses.

### rMSC culture

MSCs from rat bone marrow were cultured and prepared for transplantation as described previously [18]. The bone marrow was extracted from femurs and tibias and seeded into plastic dishes at a density of 1 × 10^6^ cells/cm^2^ in Dulbecco’s modified eagle medium-F12 containing 10% fetal bovine serum, penicillin (100 U/mL), and streptomycin (100 μg/mL) and kept at 37 °C and 5%/95% CO_2_/air. After 24 h, the dishes were washed with PBS and the medium was changed every 2–3 days. Cells were passaged at ~ 90% confluency using 0.25% Trypsin-EDTA and suspended in 0.9% NaCl containing DNAse (Ambion™ DNase I, 0.625 U/ml, all from Invitrogen Inc., Carlsbad, CA, USA) prior to administration.

### Optic-nerve injury and intraocular injections

Optic nerve crush was performed as previously described [18, 26]. Rats were anesthetized with ketamine (75 mg/Kg) and xylazine (10 mg/kg); ocular topical ointment was applied. Under a stereoscopic microscope, the optic nerve was exposed by making an incision in the skin covering the orbital bone. The nerve sheath was cut and the nerve was crushed with tweezers (Dumont #5, 45° angle, 0.05 × 0.01 mm tips, World Precision Instruments, Sarasota, FL, USA) for 15 s, at ~ 1 mm from the optic disc. Immediately after crush, 5 × 10^5^ MSC or vehicle (5–10 μL) was injected intravitreally. Animals with lens or retinal blood vessel damage were excluded. Finally, the incision was sutured. Animals were kept warm and under supervision until recovery from anesthesia.

### Visual behavior

Animals had the contralateral nerve transected 1 week before, following similar procedures used for crush, to eliminate perception by the healthy eye (Additional file 1: Figure S4, upper panel). Tests were performed at baseline (optokinetic reflex) and 70 d.a.c. The interior of the apparatus or box was cleaned between animals.

The optokinetic reflex was evaluated using an OptoMotry apparatus (Cerebral Mechanics Inc). Rats were placed on a platform surrounded by monitors showing grating in clockwise (left eye) or counterclockwise (right eye stimulation) direction. The rotation speed and spatial frequency were progressively increased from 0.042 to 0.642 cycles/degrees, at maximum contrast. A blinded observer differentiated random movements from those following the grating and repeated at least once (response). Results display the maximum frequency responded.

The looming response was analyzed as previously described [15, 28], after adapting the dimensions to rats. The rat was placed in a box (46 × 29.5 × 30.5 cm, *l* × *w* × *h*) with a shelter (20 × 15 cm at a height of 20 cm). After 5-min adaptation and when the animal moved to the center, a top monitor displayed an expanding circle, producing a shadow (Additional file 2: Video SV1). Rats were separated from the next to be tested to avoid vocalization and bias.


**Additional file 2:** Video SV1. Looming stimulus. The stimulus is given once, when the animal goes bellow the center of the monitor. (MP4 458 kb)


The white-black box was adapted from a previously described test [29]. A box (57 × 52.5 × 26.5 cm) was equally divided in (1) a “white chamber” with white inner coating in every side excluding the top, which was transparent and (2) a “black chamber” with black inner coating. An aperture (10 × 12.5 cm, *w* × *h*) allowed the animals to move between them. To check if animals had preference for the dark, which is typical in rodents, naïve animals were individually placed in the white chamber for 10 min when (a) the white chamber was light (~ 85 lx) and the black was dark (~ 1 lx) or (b) both chambers were dark. A camera with infra-red light recorded the movement of the animal. Nerve-crushed animals were tracked only in (a). Results are expressed as the fraction of the test duration spent in the black chamber.

### Light deprivation and stimulation

NGFI-A is downregulated in the absence of visual stimuli or retinocollicular connections, and it is upregulated in SC post-synaptic neurons upon *N*-methyl-d-aspartate receptors activation after glutamate release from RGCs [30–32]. Rats were placed for 24 h in the dark to downregulate NGFI-A expression and then exposed to light for 90–120 min before euthanasia to reach the peak of NGFI-A expression.

### Histological preparation and immunohistochemistry

Animals received an overdose of anesthetics and were perfused through the heart with saline and 4% paraformaldehyde. Retinas were dissected for flat mounts. The eyes, nerves, and brains were dehydrated in 10–30% sucrose and sectioned at 14–20-μm thickness. Primary antibodies used were Tuj1 (mouse, 1:250, Covance, Berkeley, CA, USA), Osteopontin (rabbit, 1:500, R&D System, Minneapolis, MN, USA), and NGFI-A (anti-egr1, rabbit, 1:400, Santa Cruz Biotechnology Inc., Santa Cruz, CA, USA). Secondary antibodies used were Cy3-conjugated goat anti-mouse or anti-rabbit IgG (1:1000, Jackson Immunoresearch Laboratories, West Grove, PA, USA) and Alexa 488- or 555-conjugated goat anti-rabbit IgG (1:1000, Invitrogen Inc.). Nuclei were counterstained with TO-PRO-3 (Invitrogen Inc.) or DAPI (4′,6-diamidino-2-phenylindole, 2.7 mg/ml, Sigma-Aldrich, St Louis, MO, USA).

### RGC survival analysis

Fifteen to 20 images of 0.05 mm^2^ were acquired using a LSM 510 microscope (Zeiss) at ~ 1.0 (central) and 3.5 mm (peripheral retina) from the optic disc, in all quadrants of the retina. Tuj1^+^ cells were counted by a blinded observer, averaged between center and periphery and normalized by control. Results are expressed as mean ± SEM. The soma areas of Tuj1+ cells were measured in the GCL of retinas stained with Tuj1 (crush) or double-stained with Tuj1 and Osteopontin antibodies (naïve). Measurements were performed in 15–20 images of each retina (*n* = 3), using ImageJ. Results are expressed as number of cells with a given soma area (μm^2^) in all images.

### Axon labeling and counting

RGC axons were anterogradely labeled by intravitreal injection of cholera toxin B conjugated to Alexa 488 or 555 (4 μl, 0.2% CTB-488 or CTB-555, Invitrogen Inc.) 2 days before euthanasia. Nerve sections were observed under an Axiovert 200 M microscope (Zeiss), using a × 40 objective lens. The center of the field was positioned from 0.25 to 2.00 mm from the proximal border of the crush site. At each distance, a blinded observer counted the number of CTB^+^ axons and measured the width of the nerve, in five longitudinal sections. We estimated the total number of axons at each distance as previously described [5]. Because there was a reduction in nerve thickness and opacity 240 d.a.c., these nerves were not sectioned but placed in 80% glycerol overnight and imaged under an LSM 710 Microscope (Zeiss).

### Quantification of NGFI-A^+^ cells

Three brain sections distributed in the rostro-caudal axis were chosen per animal. Three images (medial to lateral) of 0.135 mm^2^ covering the superficial layers of the SC were made per section, using an LSM 510 microscope (Zeiss). A blinded observer counted NGFI-A^+^ nuclei and normalized by the area.

## Results

### RGC survival

**Fig. 1:**
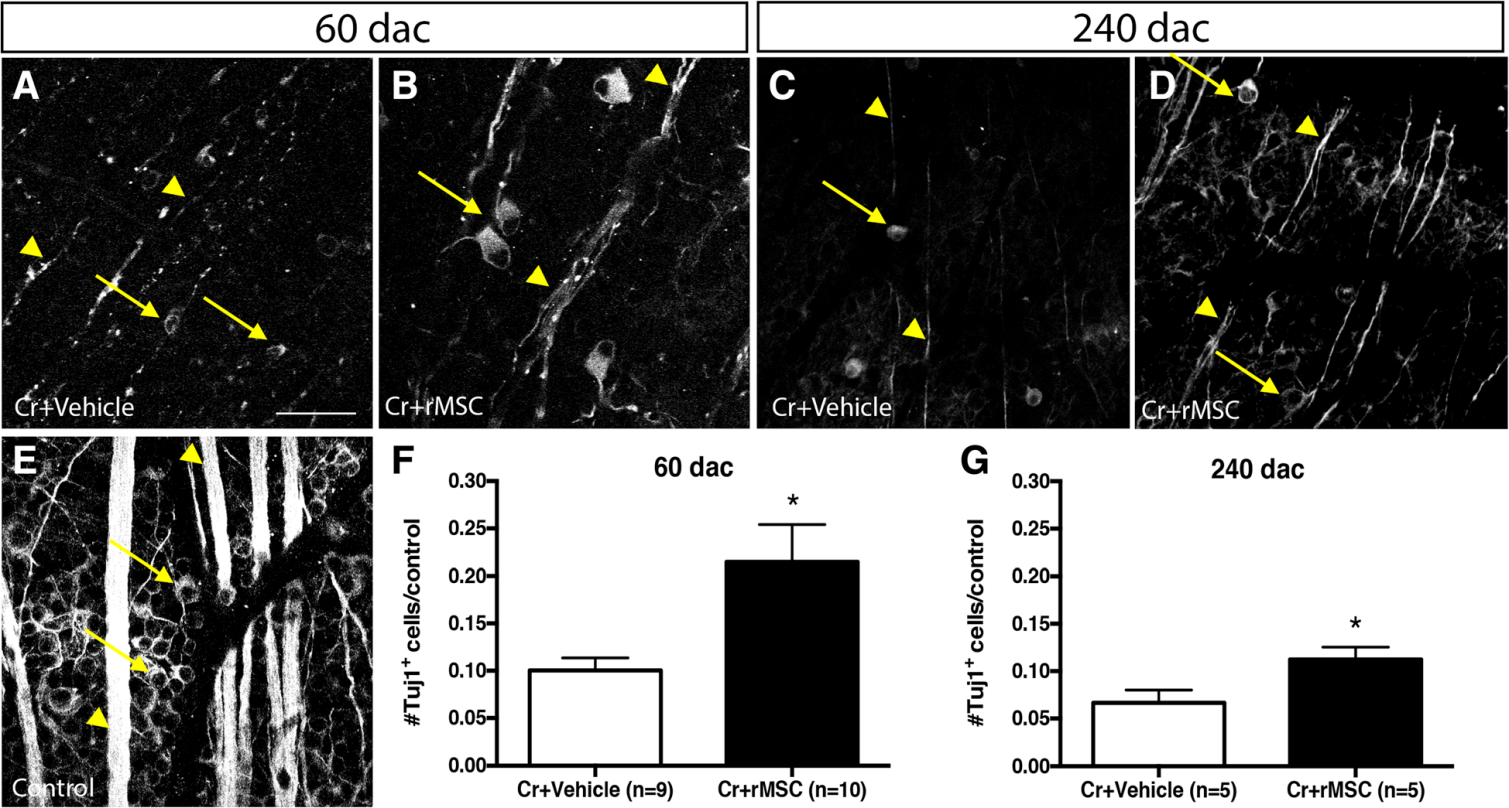
rMSCs increased RGC survival up to 240 days after crush. **a**–**e** Confocal images of flat-mounted retinas. Control retina (contralateral eye) shows numerous Tuj1^+^ cells and intact axon bundles. **a**, **c** Vehicle-injected retinas; **b**, **d** rMSC-treated retinas. Arrows point to cell bodies and arrowheads to axon bundles. **f**, **g** In rMSC-injected animals, surviving RGCs were significantly increased in both time points after crush. *P < 0.05, **P < 0.01; unpaired *t* test. Scale bar 50 μm

RGC density is normally higher in the central retina, but we observed that by 60 and 240 d.a.c. RGC density became homogeneous (*P* > 0.05, unpaired *t* test comparing the number of Tuj1^+^ cells in the central versus peripheral retina from vehicle-injected group; Additional file 1: Table S1 shows Tuj1^+^ cells per square millimeter), suggesting a greater loss of central RGCs. Retinas of vehicle-injected animals had degenerated fibers (arrowheads in Fig. 1a, c) and reduced Tuj1^+^ cells when compared to control (arrows in Fig. 1e). In rMSC-injected animals, Tuj1^+^ cells were more numerous and exuberant (arrows in Fig. 1b), while fibers were better preserved (arrowheads in Fig. 1b, d) when compared to vehicle-injected animals. Of notice, the soma area of most RGCs was equal to or smaller than 150 μm^2^ in vehicle-injected animals (Additional file 1: Figure S1E), while a considerable number of cells bigger than 150 μm^2^ was detected in rMSC-injected animals (Additional file 1: Figure S1F), suggesting that rMSCs protected RGCs belonging to a larger subtype. Similarly, the activation of mTOR protects and stimulates the regeneration of a subpopulation of large RGCs, the alpha-RGCs (αRGCs), that also express osteopontin [14]. Accordingly, we observed that the area of osteopontin^+^ cells in naïve rat retinas ranged from 150 to 500 μm2, with most cells measuring around 200–350 μm2 (Additional file 1: Figure S1A-D), which was the same area range of most of RGCs that were protected by rMSC injection after optic nerve crush. Since smaller RGCs were equally preserved after crush and vehicle/rMSC injection in all time points analyzed (Additional file 1: Figure S1G), the predominance of larger RGCs in rMSC-treated retinas indicate a possible role of rMSC therapy in αRGCs neuroprotection.

### Axonal regeneration and target reinnervation

**Fig. 2:**
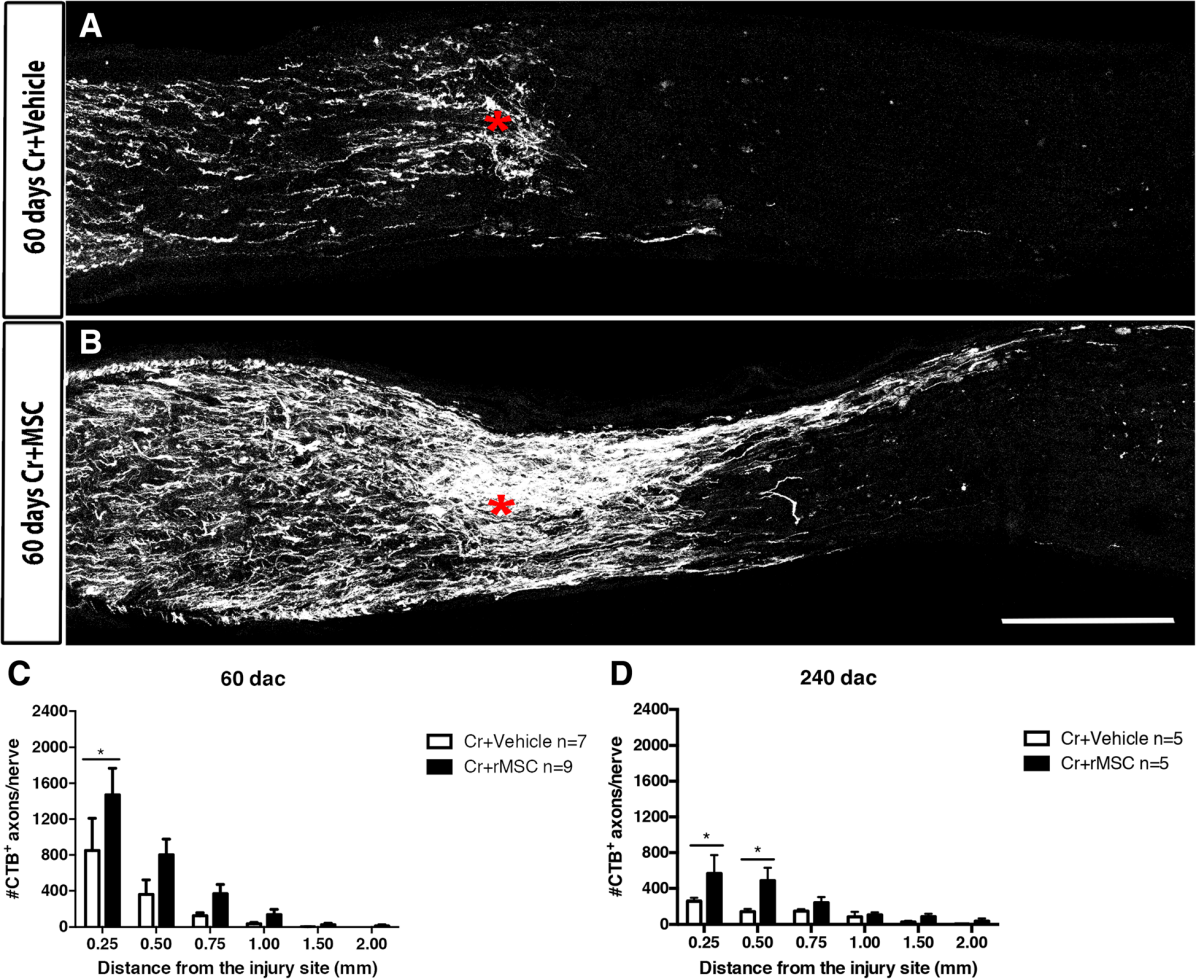
rMSCs increased axonal outgrowth up to 240 days after crush. (**a**, **b**) Photomontages of confocal images of optic nerve sections 60 d.a.c. and injection of vehicle (A) or rMSC (**b**). RGC axons were anterogradely labeled with cholera toxin (**b**) conjugated to Alexa 555 (CTB-555). The asterisk indicates the crush site. (**c**, **d**) Quantification of axons at increasing distances from the crush site at 60 (**c**) and 240 (**d**) d.a.c. *P < 0.05; two-way ANOVA with Holm-Sidak’s multiple comparisons test. Scale bar 250 μm

**Fig. 3:**
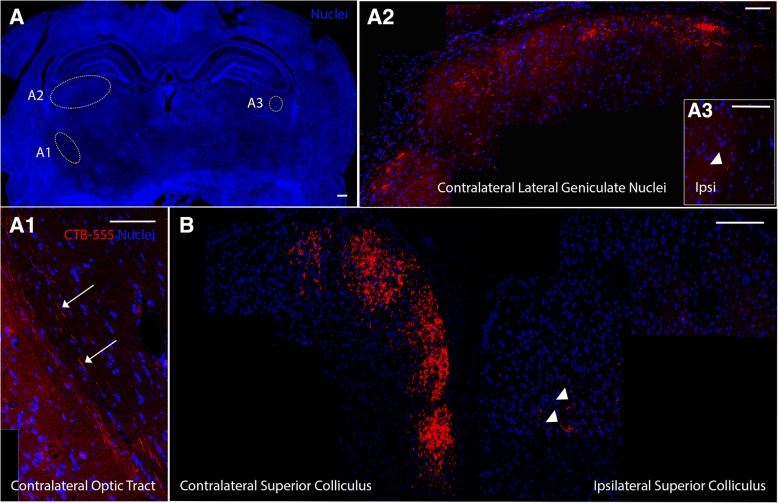
rMSC promoted target reinnervation 60 d.a.c. **A** Photomontage of a coronal brain section stained for nuclei, indicating the reinnervated areas shown in (**A1**) (optic tract, contralateral to the crushed nerve), (**A2**), and (**A3**) (lateral geniculate nuclei). RGC axons were labeled with cholera toxin (**B**) conjugated to Alexa 555 (CTB-555). **B** CTB-555^+^ axons were found at the SC. Nuclei were labeled with TOPRO-3 (blue). Scale bar: (**A**) 1000 μm; (**A1**, **A2**, **A3**, **B**) 100 μm. SC superior colliculus

Sixty d.a.c., we observed ~ 2× regenerated axons at 0.25 mm in rMSC-injected than in vehicle-injected animals (*P* < 0.05, two-way ANOVA with Holm-Sidak’s multiple comparisons test; Fig. 2a–c). At 240 d.a.c., there were fewer regenerated axons in both groups, but the rMSC-treated group had significantly more and longer axons up to 0.50 mm, indicating that the stimulus to regeneration was sustained (*P* < 0.05, two-way ANOVA with Holm-Sidak’s multiple comparisons test; Fig. 2d). One rMSC-injected animal showed a cluster of CTB^+^ axons crossing the crush site through the periphery and traveling through the center of the mid/distal nerve (arrows in Additional file 1: Figure S2A’); the optic chiasm showed several axons traveling in the ipsilateral or contralateral direction (arrows in Additional file 1: Figure S2B). CTB^+^ axons reached the optic tract (Fig. 3A1), lateral geniculate nuclei (Fig. 3 A2), and superior colliculus (SC, Fig. 3B). CTB was predominant in the contralateral side and discrete in the ipsilateral side (arrowheads in Fig. 3A3 and B), indicating that most regenerated axons crossed the optic chiasm and that a small fraction proceeded without crossing it.

### Synaptic reconnection

**Fig. 4:**
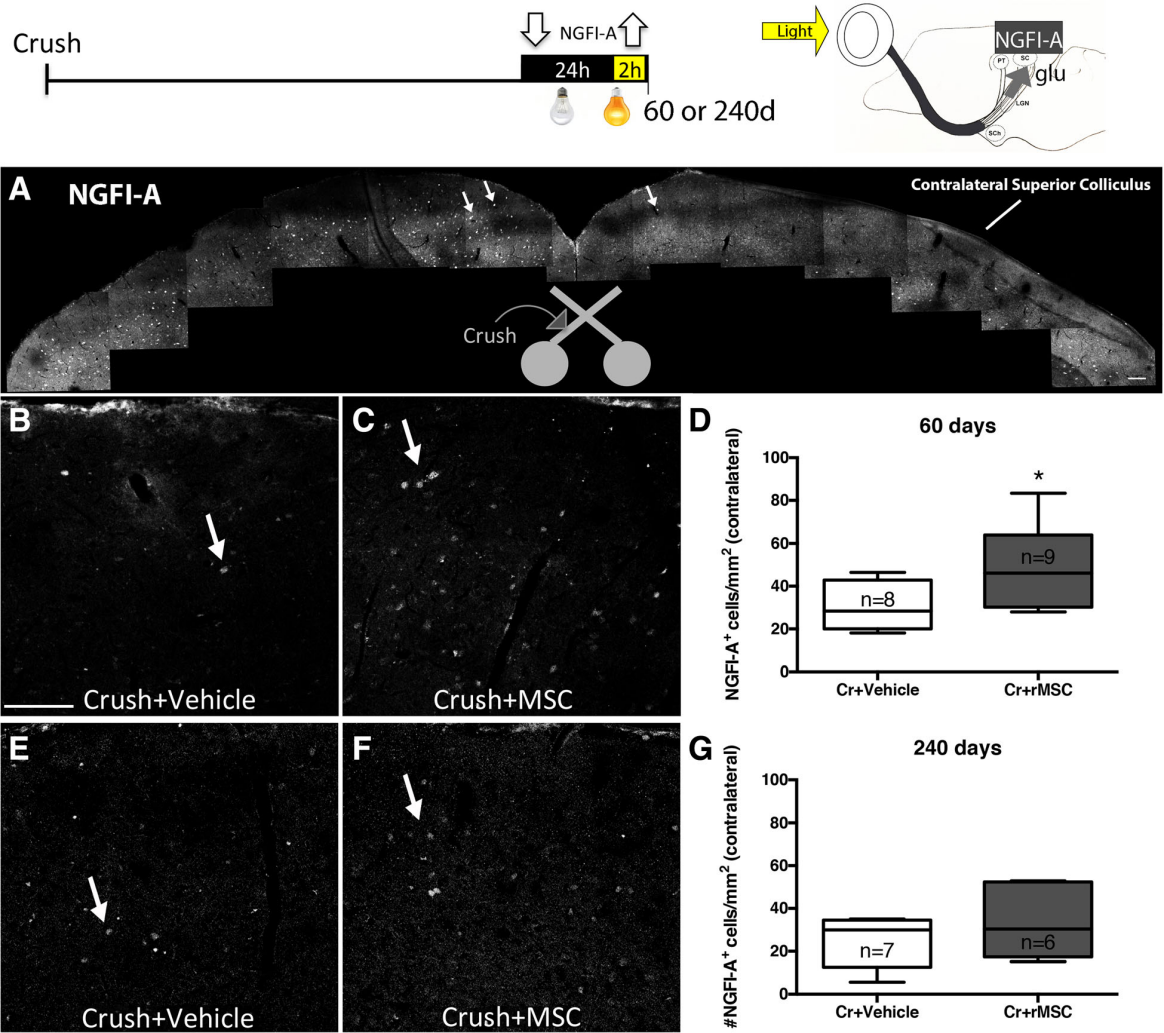
rMSC promoted synaptic reconnection in the SC. Upper panel shows the experimental design and scheme indicating that RGCs glutamatergic synapses in response to light stimulus lead to the expression of NGFI-A by post-synaptic neurons at the SC. **a** Photomontage of confocal images of the SC 60 d.a.c. NGFI-A+ cells (arrows) were abundant in the crushed-nerve-ipsilateral side and rare in the contralateral side. (**b**, **c**) NGFI-A+ cells in the contralateral SC 60 (**b**, **c**) and 240 (**e**, **f**) d.a.c. NGFI-A+ cells were increased in the contralateral SC of rMSC-treated animals 60 days (**d**) but not 240 days (**g**) after optic nerve crush. **P < 0.01 (Mann-Whitney test). Scale bar, 100 μm. SC: superior colliculus, PT: pretectum, LGN: lateral geniculate nucleus, SCh: suprachiasmatic nucleus, glu: glutamate

Animals were submitted to light deprivation/stimulation, and their SC was analyzed (Fig. 4, upper panel). The hemisphere ipsilateral to crush had several NGFI-A^+^ cells (arrows in Fig. 4a), while only few cells were seen in the contralateral side, indicating massive loss of RGC connections from the crushed nerve. There were significantly more NGFI-A^+^ cells in the contralateral SC of rMSC-injected than vehicle-injected animals 60 d.a.c. (*P* < 0.05, Mann-Whitney test; Fig. 4b–d), indicating that rMSC promoted RGC reconnection to the brain.

**Fig. 5:**
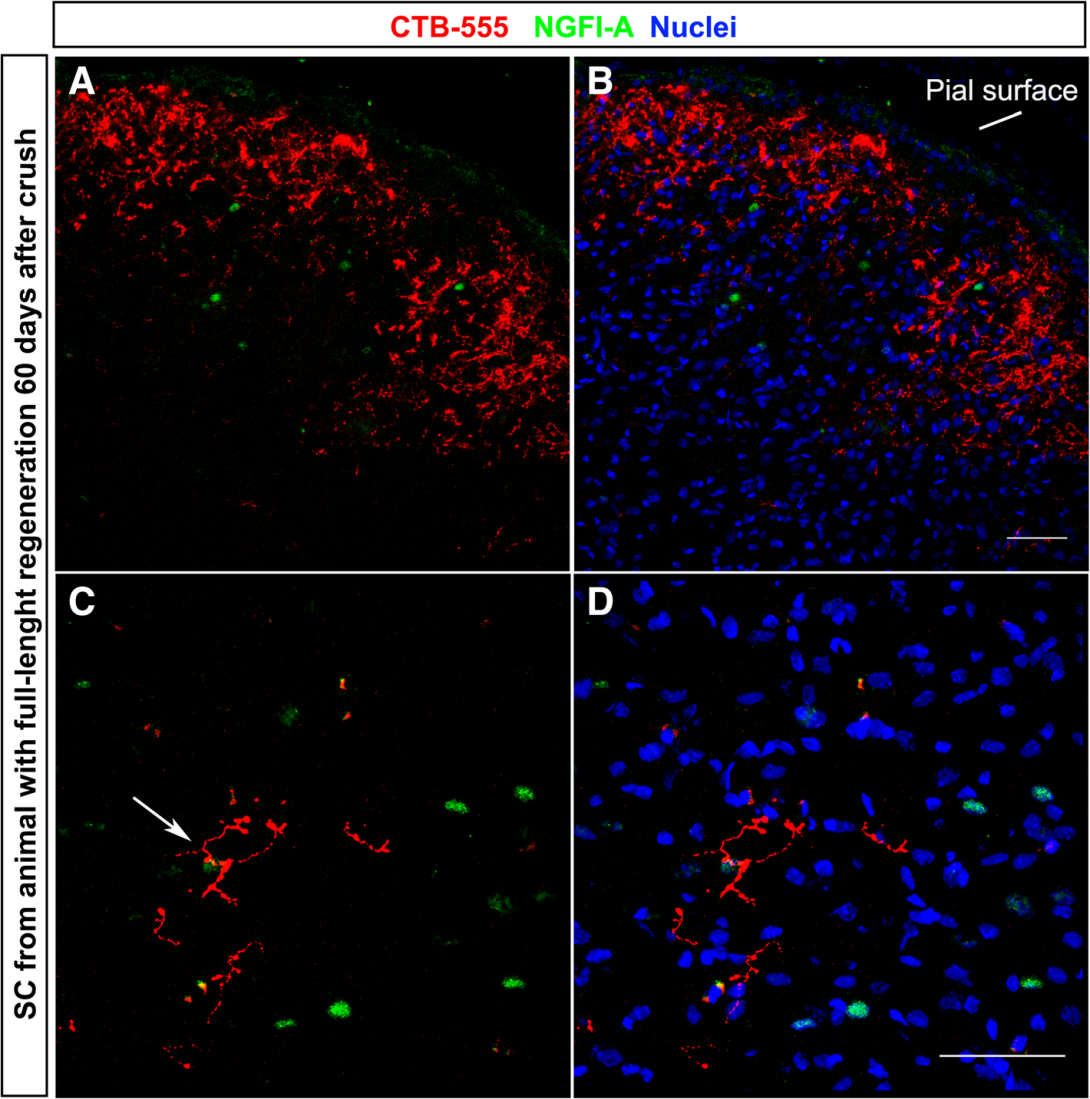
Axon regeneration and NGFI-A expression. **a**-**d** NGFI-A expression in the reinnervated area of the SC. Some CTB-555^+^ axons were found near NGFI-A^+^ cells (arrow in **c**). Nuclei were labeled with TOPRO-3 (blue). Scale bar, 50 μm. SC superior colliculus

To provide additional evidence that NGFI-A expression was triggered by RGC regenerated axons, NGFI-A was analyzed in animals with CTB-555-labeled axons. Figure 5a, b shows CTB-555^+^ axons (red) in the SC and a few NGFI-A^+^ nuclei (green) in the reinnervated area. Axon terminals are seen near an NGFI-A^+^ cell (arrow in Fig. 5c), suggesting that a synapse was made between the CTB-555^+^ terminal and the NGFI-A^+^ neuron.

However, even in animals in which CTB^+^ axons were not found, NGFI-A^+^ cells were observed in both superior colliculi (Fig. 4a). One possible explanation is that a small number of regenerated axons are hardly observable in coronal brain sections. A second explanation is the presence of ipsilateral projections from the healthy eye. To investigate that, we injected CTB CTB-555 in the left (crushed-nerve) and CTB-488 in the right (uncrushed-nerve) eyes. Additional file 1: Figure S3 shows the contralateral SC of a vehicle-injected animal 60 d.a.c. Several CTB-488^+^ axons (green) derived from the uncrushed nerve were found. NGFI-A^+^ cells (magenta) were found near these axons (arrows in Additional file 1: Figure S3), suggesting that ipsilateral projections are responsible for the presence of NGFI-A^+^ cells in the absence of regeneration of the contralateral nerve.

### Visual behavior analysis

Before crush, the optokinetic response was observed in all animals, but in none of the animals at 70 d.a.c. (Additional file 1: Figure S4A-B). Similarly, while naïve animals responded to the looming stimulus by either freezing or running (Additional file 3: Video SV2), none of the nerve-crushed animals did (Additional file 4: Video SV3, Additional file 1: Figure S4D). Finally, naïve animals preferred the dark chamber when allowed to move between light and dark environments but randomly chose a chamber when both environments were dark (light versus dark: *P* < 0.01; dark versus dark: *P* = 0.0015, two-way ANOVA with Holm-Sidak’s multiple comparisons test; Additional file 1: Figure S4F, upper graph). While naïve animals spent 86% of the time in the dark (0.8600 ± 0.06859), nerve-crushed animals stayed less than 50% of the time in the dark, indicating loss of light perception (Additional file 1: Figure S4F, bottom graph). Time spent in the dark was not significantly different when comparing vehicle (0.4196 ± 0.1828) and rMSC (0.1933 ± 0.07330) groups (*P* = 0.3084), but it was significantly higher in naïve animals (*P* < 0.05 when compared to vehicle injection, *P* < 0.001 when compared to rMSC injection, one-way ANOVA with Tukey’s multiple comparisons test, Additional file 1: Figure S4F, bottom graph).


**Additional file 3:** Video SV2. Naïve animal response to looming stimulus. The animal perceives the stimulus and runs to hide down the shelter (upper right corner). (MP4 10503 kb)



**Additional file 4:** Video SV3. Nerve-crushed animal response to looming stimulus. The animal does not perceive the stimulus and does not change its behavior. (MP4 10394 kb)


### Axonal and synaptic degeneration long-term after crush

**Fig. 6:**
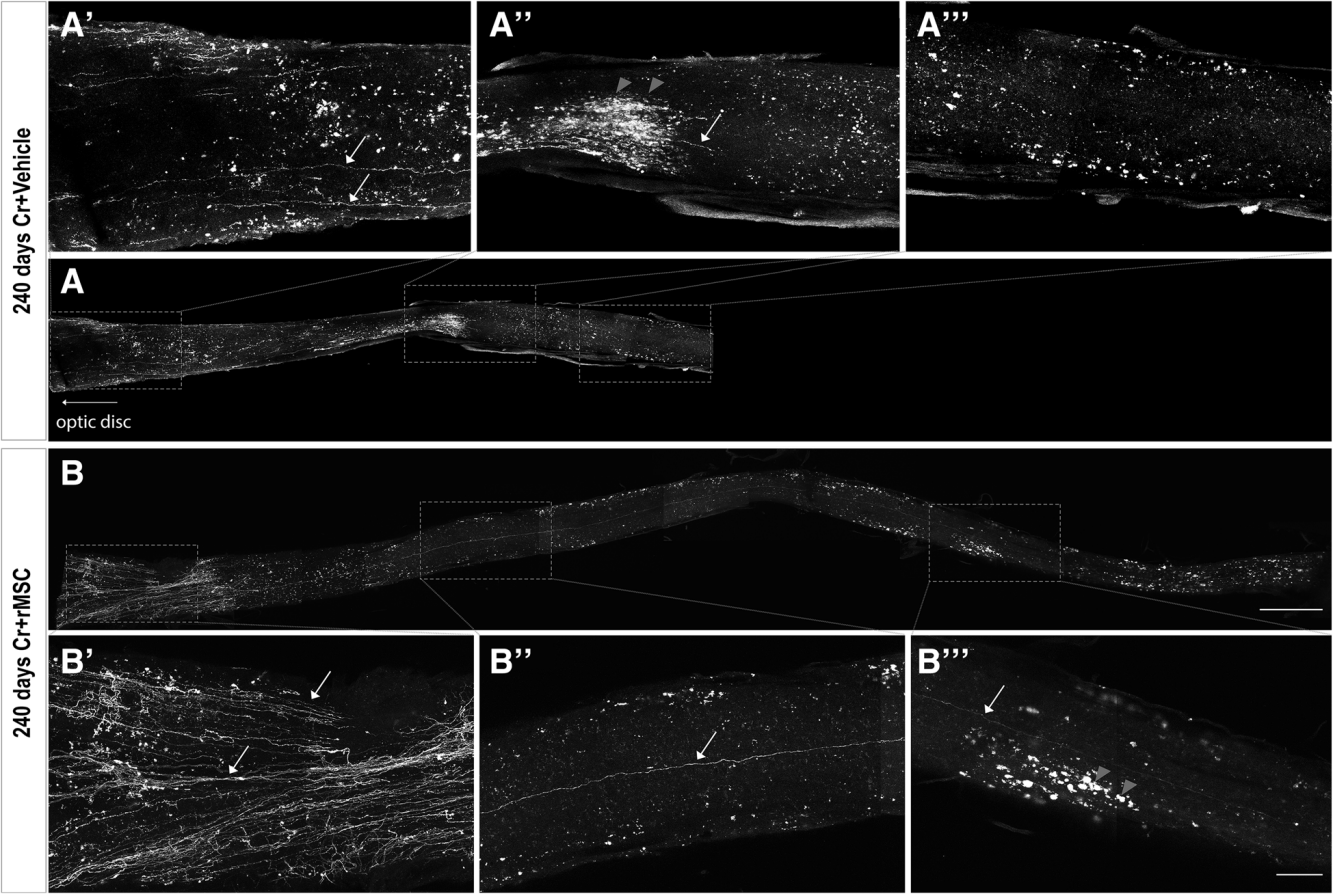
RGC axons 240 days after crush. **A**, **B** Photomontage of confocal images of the optic nerve of vehicle- (**A**) or rMSC-injected (**B**) animals. In the vehicle-injected animal whose nerve is shown in (**A**), only a few CTB-555^+^ axons (white) were seen in the proximal part of the nerve (arrows in **A′**), with rare axons extending shorter distances (arrow in **A″**). No axons were found in the distal portion of the nerve (**A′′′**). In the rMSC-injected animal whose nerve is shown in (**B**), more axons were seen in the proximal part of the nerve (arrows in **B′**), with rare axons along the nerve (arrow in **B″**) and reaching distal portion (arrow in **B′′′**). Gray arrowheads show autofluorescent signal in both nerves (**A″**, **B′′′**). Scale bar: (**B**) 500 μm; (**B′′′**) 100 μm

Possible explanations for the absent functional recovery may be the insufficient number of reconnected axons, lack of myelination, incorrect targeting and instability of the new synapses. Indeed, optic nerves were thinner and almost transparent 240 d.a.c. (data not shown), suggesting demyelination, while the analysis of the whole nerve indicated fewer axons than in sections done 60 d.a.c. (compare Fig. 6A′ to Fig. 2a and Fig. 6B′ to Fig. 2b), with rare axons seen in the vehicle-injected animals (arrows in Fig. 6A′ and A′′), suggesting axon degeneration in both groups. In rMSC-injected animals, more axons were observed (Fig. 6B, B′), as confirmed by the quantification shown in the Fig. 2D, with few axons extending full-length along the nerve (arrows in Fig. 6B′ and B″). In addition, the number of NGFI-A^+^ cells became equivalent in both crushed groups 240 d.a.c. (Fig. 4g), suggesting that, like axons, synapses degenerated over time.

## Discussion

Bone marrow cells have been tested in several animal models of glaucoma. Most studies analyzed RGC survival up to 4 weeks after induction and concluded that MSC have a neuroprotective and regenerative effect [33–37]. Mead and coworkers observed that MSC promoted a ~ 2-fold increase in RGC survival 3 weeks after optic nerve crush [38], which is consistent with our previous results [18]. In this study, we present a further analysis on the long-term effects of rMSC therapy and we show sustained neuroprotection and axon regeneration. Interestingly, a recent study analyzed the effects of human Wharton Jelly’s injected intravitreally and found that RGC neuroprotection was lost between 14 and 30 days after optic nerve crush [39], while in our observations RGC neuroprotection is sustained up to 240 days. However, the number of MSC injected in that study was × 25 smaller than the number we injected in the present study. As we demonstrated that with our approach, the injected cells remain for at least 18 weeks in the eye [18], we associate our prolonged effect with both dosage and prolonged time that cells remain for in the site of injection.

The current view is that MSCs can be neuroprotective and pro-regenerative because they secrete soluble factors [19, 38, 40] or vesicles containing a variety of molecules—ranging from miRNA to trophic factors—that can be internalized by host cells [41–43]. Many studies have associated MSC effects with increased neurotrophins [44, 45], and many of them have suggested that MSC can control inflammation [46], e.g., by promoting an alternative microglial activation [47, 48] or modulating the inflammatory infiltrate [49], which may create a growth-permissive environment [50]. Indeed, RGCs were capable of extending more axons in rMSC-injected animals and regenerated axons were observed up to the brain. The persistence of rMSCs in the eye [18] could explain the sustained effects.

NGFI-A expression was doubled in rMSC-treated animals, while BMMCs promoted ~ 1.5-fold increase [17], suggesting that rMSCs are equivalent or more efficient than BMMCs. The detection of single or few axons by anterograde labeling and optical microscopy is limited by RGC uptake of the tracer, tissue sectioning, and optical resolution, what may explain why not all animals with increased NGFI-A^+^ cells had CTB^+^ axons in the brain. In addition, the efficiency of tracing may be impacted by deficient tracer transport in axons that are damaged although structurally connected, suggesting that at least part of the regenerated axons might have not been traced by our methods [51].

Long distance endogenous axon regeneration and target reconnection has been demonstrated in rodents but, to our knowledge, we first demonstrate it after cell therapy, with exception of our study using BMMCs [17]. It has been shown that, in mice, regeneration is often limited to larger RGCs belonging to the alpha subtype [14]. Since larger RGCs were also the prevalent type of protected RGCs in rMSC-treated animals, our results suggest that αRGCs were preferentially protected by rMSC therapy. Since in previous studies a robust regeneration of αRGCs was obtained after activation of mTOR pathway through PTEN deletion [14, 52], our results suggest a similarity of mechanisms of MSC therapy and genetic modulation of the RGC growth program through activation of mTOR. It is possible that factors secreted by MSCs modulate this pathway in RGCs. This is consistent with MSC production and release of regulatory RNA such as microRNAs inside extracellular vesicles [43]. Overall, our results are comparable to studies that used complex approaches involving transgenic mice [9], inflammatory stimulation [12], and/or increase of electrical activity [15]. These studies showed axon regeneration in mice that have a shorter optic pathway than rats, with varied levels of target reconnection and partial recovery of the visual function.

Although we cannot completely exclude the presence of spared axons, we used several criteria to identify regenerated axons. For instance, in our previous analyses shortly after injury (1, 14, and 28 days), there were no long-projecting RGC axons, suggesting consistency of our methods to crush the entire optic nerve [17, 18, 25–27]. Furthermore, regenerated axons in this study contoured the crush site, possibly avoiding the glial scar, and then proceeded in a non-linear fashion through the center of the nerve until the chiasm, where they were scattered and with some degree of turning, different from the typical linear orientation of spared axons along the nerve [53].

Although rMSC promoted target reconnection by 60 d.a.c., there was a decline in the number of regenerating axons and retinocollicular synapses by 240 d.a.c., demonstrating loss of effect. Unstable synapses formed by 60 d.a.c. might degenerate, what could explain the absence of functional recovery 70 d.a.c. Also, more regenerating RGCs may be necessary to recover a vision-dependent behavior, and target recognition failure and dysmyelination are major obstacles to functional recovery [52]. Therefore, combinatorial approaches and oligodendroglia-targeted therapies are necessary to overcome multiple obstacles for optic nerve regeneration [54]. In addition, the permanence of MSCs in the eye may obstruct the passage of light, and other potential concerns to cell therapy were not addressed in this study.

## Conclusions

The intravitreal injection of rMSCs following optic nerve crush promoted sustained RGC neuroprotection and long-distance regeneration, with transient target reconnection. Since we have previously described long-term permanence of MSCs in the eye [18], the progressive loss of the axon regenerative effect seen in this study may not be solely attributed to the clearance of MSCs but also to a limitation of cell therapy alone in achieving permanent neuronal reconnection to its targets. For instance, the lack of visual behavior recovery indicates that RGCs need additional stimuli to make stable reconnections. Nevertheless, our results with MSC therapy indicated a robust, sustained neuroprotective effect up to the longest time point analyzed. Further studies to better elucidate MSC mechanisms will enable approaches without the injection of cells but of molecules directed to MSC original targets for optimization and translational purposes. Our study suggests that the combination of MSCs or of its secretome with additional therapeutic approaches is more likely to sustain therapeutic effects for longer time points.

## Additional files

Additional file 1 **:** **Table S1.** Number of Tuj1-positive cells in the retina. Number of cells per square millimeter of retina, SEM, and number of experiments (*n*). **Figure S1.** Distribution of surviving RGCs according to cell soma area. **Figure S1.** Distribution of surviving RGCs according to cell soma area. A-C: Confocal images of naïve flat-mounted retinas stained for Tuj1 (A), osteopontin (OPN) (B) and the merge (C). D: Distribution of Tuj1-OPN+ cells in the ganglion cell layer of the retina according to the cell soma area. E-F: Distribution of Tuj1+cells soma area in vehicle-injected (E) and rMSC-injected (F) groups 60 d.a.c. Dashed line represents the minimum area of OPN+cells. G: Survival of sizes-subtypes of TUJ+ cells at different time-points after injury, showing that rMSC-therapy increases preferably survival of RGCs with area greater than 150 μm^2^. Scale bar: 50 μm. **Figure S2.** Long-distance regeneration of RGC axons 60 days after crush and rMSC treatment. A: Photomontage of confocal images of several sections of the optic nerve. CTB-555^+^ axons (white) were found in the middle of the nerve (A’) and just before the optic chiasm (A′′). C: Axons were seen crossing the chiasm to both contralateral and ipsilateral hemispheres. Scale bar: A: 250 μm; A′, A′′ B: 50 μm. **Figure S3.** Ipsilateral axons and NGFI-A expression. Left panel shows the experimental design. Regenerated CTB-555^+^ axons (red) were not found in the SC of vehicle-injected animals. CTB-488^+^ axons (green) from uncrushed nerve were found in the crushed-nerve contralateral superior colliculus, near NGFI-A^+^ cells (magenta). Some axons are seen near NGFI-A^+^ cells (arrows). Nuclei were labeled with TOPRO-3 (blue). Scale bar: 50 μm. SC: superior colliculus. **Figure S4.** Visual behaviors analysis. After 63 days of left-nerve crush, animals were submitted to axotomy of the right nerve and tested for visual behaviors after 1 week (day 70). A-B: Optokinetic reflex; most animals responded up to the highest frequency before crush but none of the nerve-crushed animals recovered the reflex. C-D: Looming response was observed in naïve animals after but not after crush; E-F: when placed in a box with light and dark chambers, naïve animals spent most of the time in the dark. When both chambers were in the dark, they did not show any preference to one or the other side of the box. Total test duration per animal was of 600 s. **P < 0.01 (two-way ANOVA with Holm-Sidak’s multiple comparisons test). Bottom graph: Naïve animals spent most of the time in the dark, while nerve-crushed animals spent half or less than half of the time in the dark, without significant differences between vehicle and rMSC-injected groups. *P < 0.05; ***P < 0.001; One-Way ANOVA with Tukey’s multiple comparisons test. (DOCX 5530 kb)

## Abbreviations

BMMCs: Bone marrow mononuclear cells
CNS: Central nervous system
CTB-488: Cholera toxin B conjugated to Alexa 488
CTB-555: Cholera toxin B conjugated to Alexa 555
d.a.c.: Days after crush
KLF: Kruppel-like family
MSCs: Mesenchymal stem cells
mTOR: Mammalian target of rapamycin
NGFI-A: Nerve growth factor-induced gene A
PTEN: Phosphatase and tensin homolog
RGCs: Retinal ganglion cells
rMSCs: Rat mesenchymal stem cells
SC: Superior colliculus
SOCS3: Suppressor of cytokine signaling 3
