# Supplementary material for: Long-term neuronal survival, regeneration, and transient target reconnection after optic nerve crush and mesenchymal stem cell transplantation
Source: Stem Cell Res Ther. 2019 Apr 17;10:121. doi: 10.1186/s13287-019-1226-9 (PMC6472105; doi:10.1186/s13287-019-1226-9)
Supplement: Supplementary file 4 — : Table S1. Number of Tuj1-positive cells in the retina. Number of cells per square millimeter of retina, SEM, and number of experiments (n). Figure S1. Distribution of surviving RGCs according to cell soma area. Figure S1. Distribution of surviving RGCs according to cell soma area. A-C: Confocal images of naïve flat-mounted retinas stained for Tuj1 (A), osteopontin (OPN) (B) and the merge (C). D: Distribution of Tuj1-OPN+ cells in the ganglion cell layer of the retina according to the cell soma area. E-F: Distribution of Tuj1+cells soma area in vehicle-injected (E) and rMSC-injected (F) groups 60 d.a.c. Dashed line represents the minimum area of OPN+cells. G: Survival of sizes-subtypes of TUJ+ cells at different time-points after injury, showing that rMSC-therapy increases preferably survival of RGCs with area greater than 150 μm2. Scale bar: 50 μm. Figure S2. Long-distance regeneration of RGC axons 60 days after crush and rMSC treatment. A: Photomontage of confocal images of several sections of the optic nerve. CTB-555+ axons (white) were found in the middle of the nerve (A’) and just before the optic chiasm (A′′). C: Axons were seen crossing the chiasm to both contralateral and ipsilateral hemispheres. Scale bar: A: 250 μm; A′, A′′ B: 50 μm. Figure S3. Ipsilateral axons and NGFI-A expression. Left panel shows the experimental design. Regenerated CTB-555+ axons (red) were not found in the SC of vehicle-injected animals. CTB-488+ axons (green) from uncrushed nerve were found in the crushed-nerve contralateral superior colliculus, near NGFI-A+ cells (magenta). Some axons are seen near NGFI-A+ cells (arrows). Nuclei were labeled with TOPRO-3 (blue). Scale bar: 50 μm. SC: superior colliculus. Figure S4. Visual behaviors analysis. After 63 days of left-nerve crush, animals were submitted to axotomy of the right nerve and tested for visual behaviors after 1 week (day 70). A-B: Optokinetic reflex; most animals responded up to the highest frequency before crush but none o [file 13287_2019_1226_MOESM1_ESM.docx]

**Table S1.**

| **60 days after crush** | |  |  |  |  |  |  |  |  |
| --- | --- | --- | --- | --- | --- | --- | --- | --- | --- |
|  | **Control (contralateral eye)** | | | **Cr+Vehicle** | | | **Cr+MSC** | | |
| **1.0 mm** | cells/mm^2^ | SEM | n | cells/mm^2^ | SEM | n | cells/mm^2^ | SEM | n |
|  | 1095 | 75.54 | 16 | 88.01 | 11.92 | 9 | 210.4 | 39.30 | 10 |
|  |  |  |  |  |  |  |  |  |  |
| **3.5 mm** | cells/mm^2^ | SEM | n | cells/mm^2^ | SEM | n | cells/mm^2^ | SEM | n |
|  | 855.1 | 66.20 | 16 | 107.5 | 15.78 | 9 | 208.2 | 38.58 | 10 |
|  |  |  |  |  |  |  |  |  |  |
| **Mean of 1.0 and 3.5 mm** | cells/mm^2^ | SEM | n | cells/mm^2^ | SEM | n | cells/mm^2^ | SEM | n |
|  | 975.1 | 65.39 | 16 | 97.76 | 13.12 | 9 | 209.3 | 38.54 | 10 |
|  |  |  |  |  |  |  |  |  |  |
| **240 days after crush** | |  |  |  |  |  |  |  |  |
|  | **Control (contralateral eye)** | | | **Cr+Vehicle** | | | **Cr+MSC** | | |
| **1.0 mm** | cells/mm^2^ | SEM | n | cells/mm^2^ | SEM | n | cells/mm^2^ | SEM | n |
|  | 1163 | 73.57 | 10 | 72.87 | 6.630 | 5 | 132.3 | 15.46 | 5 |
|  |  |  |  |  |  |  |  |  |  |
| **3.5 mm** | cells/mm^2^ | SEM | n | cells/mm^2^ | SEM | n | cells/mm^2^ | SEM | n |
|  | 965.0 | 35.96 | 10 | 69.16 | 22.93 | 5 | 107.0 | 14.36 | 5 |
|  |  |  |  |  |  |  |  |  |  |
| **Mean of 1.0 and 3.5 mm** | cells/mm^2^ | SEM | n | cells/mm^2^ | SEM | n | cells/mm^2^ | SEM | n |
|  | 1064 | 46.56 | 10 | 71.01 | 14.23 | 5 | 119.6 | 13.81 | 5 |

**Table S1. Number of Tuj1-positive cells in the retina.** Number of cells per square millimeter of retina, SEM, and number of experiments (n).


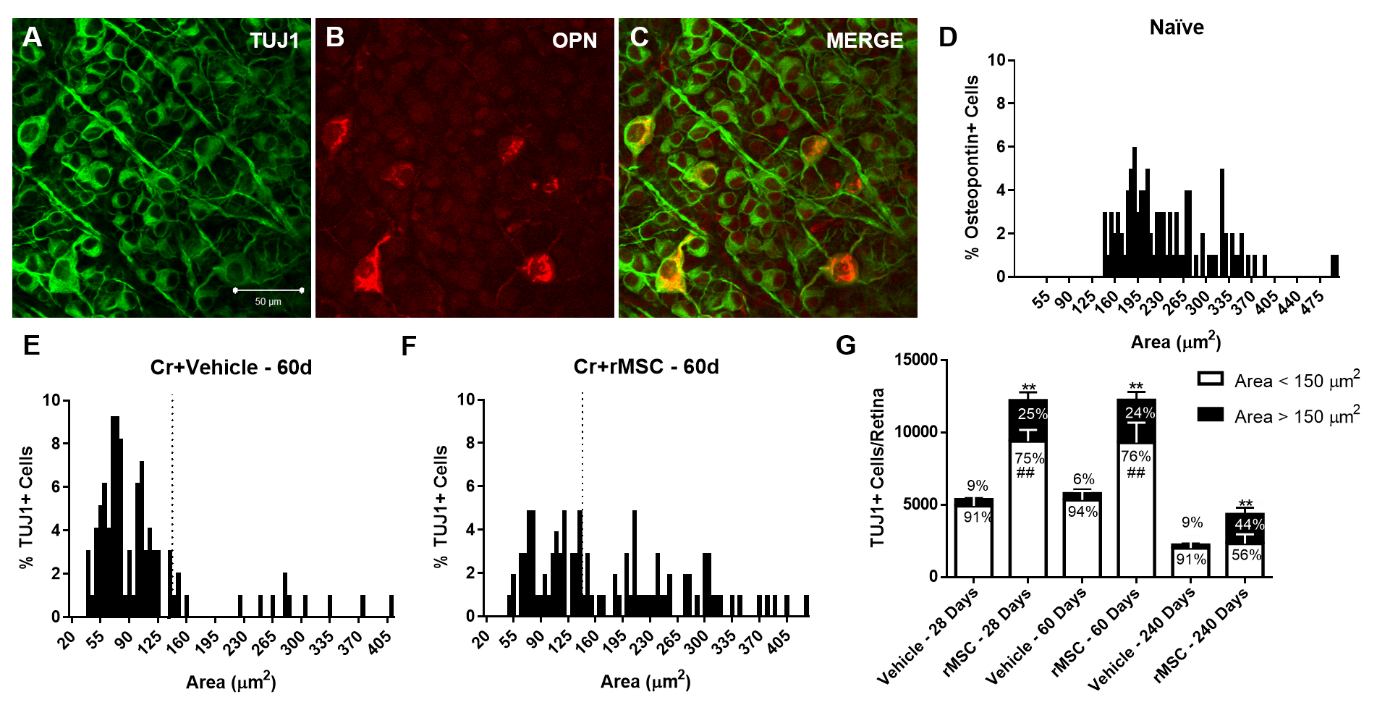


**Figure S1. Distribution of surviving RGCs according to cell soma area. Figure S1. Distribution of surviving RGCs according to cell soma area.** A-C: Confocal images of naïve flat-mounted retinas stained for Tuj1 (A), osteopontin (OPN) (B) and the merge (C). D: Distribution of Tuj1-OPN+ cells in the ganglion cell layer of the retina according to the cell soma area. E-F: Distribution of Tuj1+cells soma area in vehicle-injected (E) and rMSC-injected (F) groups 60 d.a.c. Dashed line represents the minimum area of OPN+cells. G: Survival of sizes-subtypes of TUJ+ cells at different time-points after injury, showing that rMSC-therapy increases preferably survival of RGCs with area greater than 150 µm^2^. Scale bar: 50 μm.


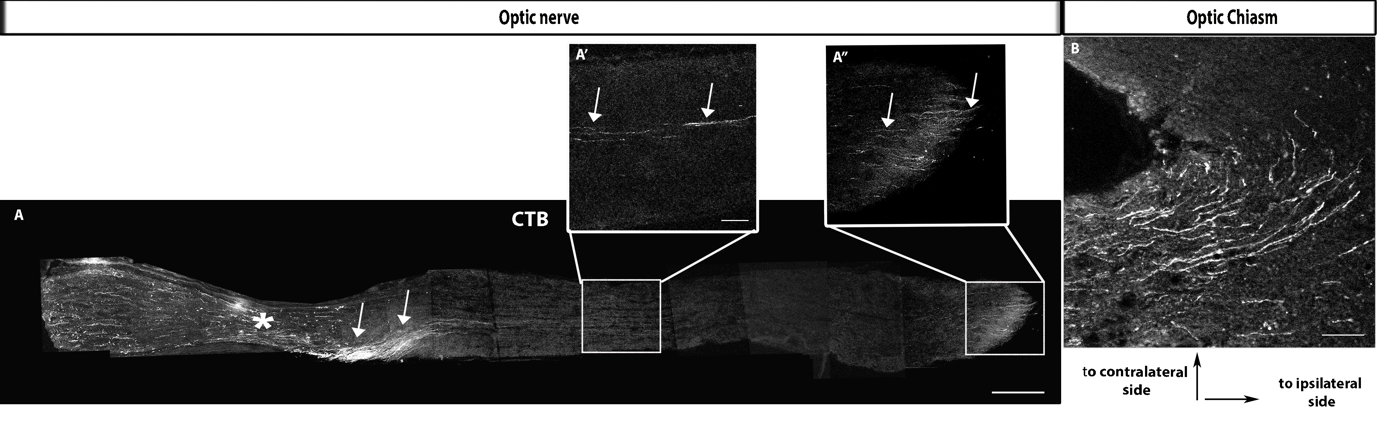


**Figure S2. Long-distance regeneration of RGC axons 60 days after crush and rMSC treatment.** A: Photomontage of confocal images of several sections of the optic nerve. CTB-555^+^ axons (white) were found in the middle of the nerve (A') and just before the optic chiasm (A''). C: Axons were seen crossing the chiasm to both contralateral and ipsilateral hemispheres. Scale bar: A: 250 μm; A', A'', B: 50 μm.

**
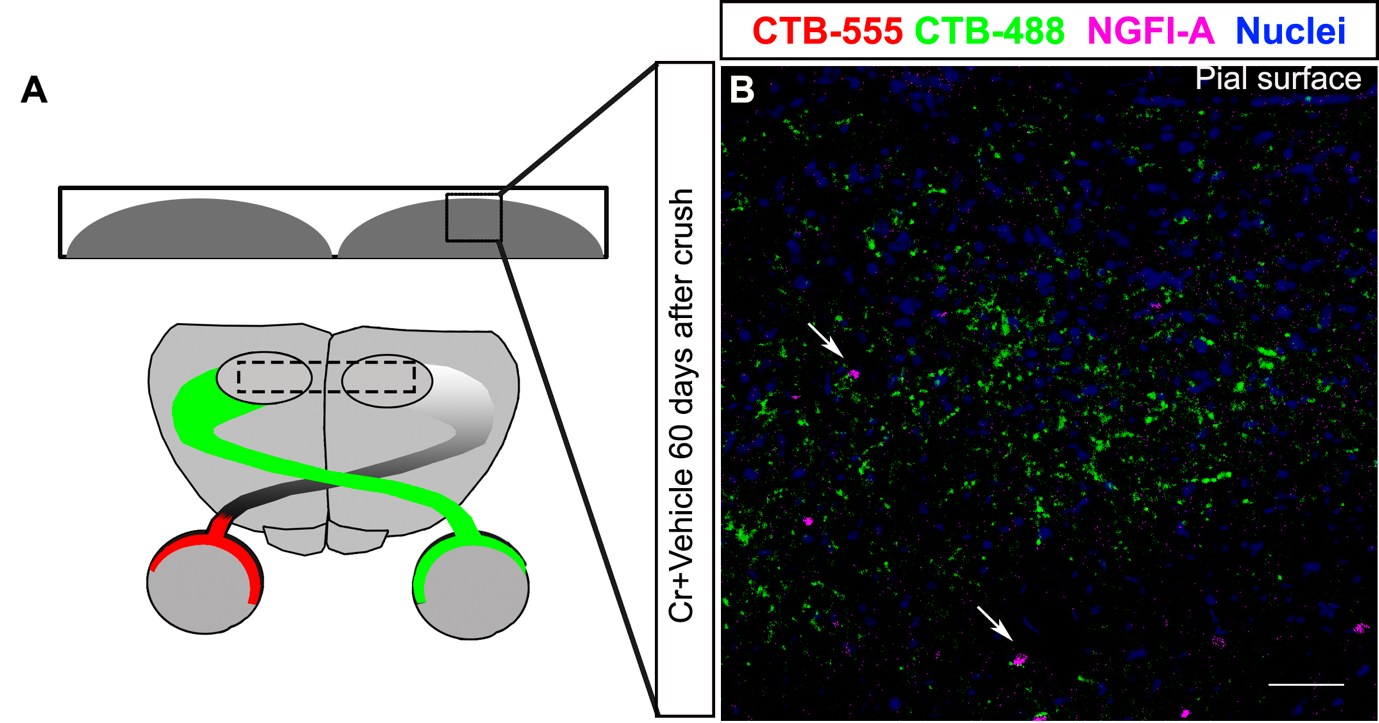
Figure S3. Ipsilateral axons and NGFI-A expression.** Left panel shows the experimental design. Regenerated CTB-555^+^ axons (red) were not found in the SC of vehicle-injected animals. CTB-488^+^ axons (green) from uncrushed nerve were found in the crushed-nerve contralateral superior colliculus, near NGFI-A^+^ cells (magenta). Some axons are seen near NGFI-A^+^ cells (arrows). Nuclei were labeled with TOPRO-3 (blue). Scale bar: 50 μm. SC: superior colliculus.

**
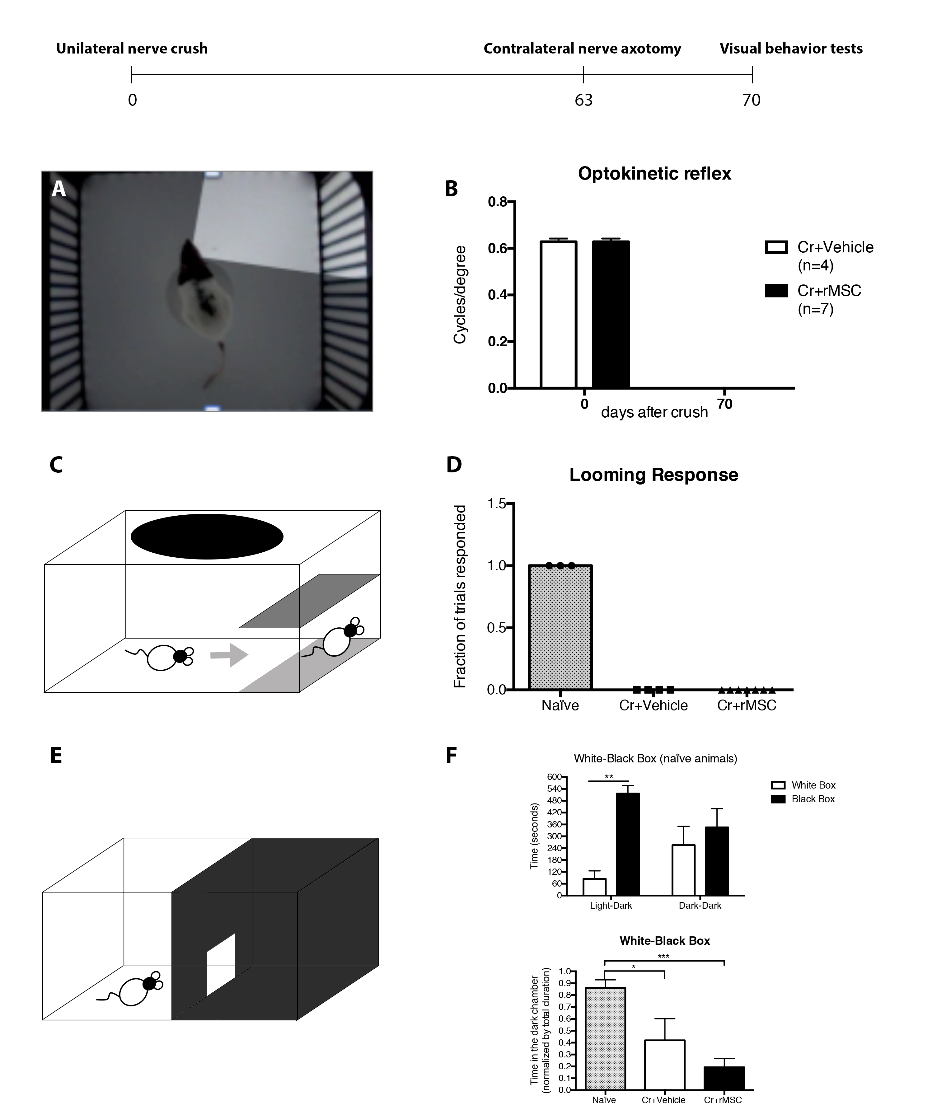
**

**Figure S4. Visual behaviors analysis.** After 63 days of left-nerve crush, animals were submitted to axotomy of the right nerve and tested for visual behaviors after 1 week (day 70). A-B: Optokinetic reflex; most animals responded up to the highest frequency before crush but none of the nerve-crushed animals recovered the reflex. C-D: Looming response was observed in naïve animals after but not after crush; E-F: when placed in a box with light and dark chambers, naïve animals spent most of the time in the dark. When both chambers were in the dark, they did not show any preference to one or the other side of the box. Total test duration per animal was of 600 seconds. **P<0.01 (Two-Way ANOVA with Holm-Sidak’s multiple comparisons test). Bottom graph: Naïve animals spent most of the time in the dark, while nerve-crushed animals spent half or less than half of the time in the dark, without significant differences between vehicle and rMSC-injected groups. *P<0.05; ***P<0.001; One-Way ANOVA with Tukey’s multiple comparisons test.
